# Acupoint massage: a comprehensive descriptive review of its forms, applications, and underlying mechanisms

**DOI:** 10.1186/s13020-025-01105-1

**Published:** 2025-04-23

**Authors:** Chongjie Yao, Xinyu Zeng, Shuaipan Zhang, Bin Xiao, Pingping Sun, Lingjun Kong, Jiming Tao, Min Fang

**Affiliations:** 1 Shuguang Hospital, Shanghai University of Traditional Chinese Medicine, Shanghai, 201203 People’s Republic of China; 2 School of Acupuncture-Moxibustion and Tuina, Shanghai University of Traditional Chinese Medicine, Shanghai, 201203 People’s Republic of China; 3 School of Rehabilitation Science, Shanghai University of Traditional Chinese Medicine, Shanghai, 201203 People’s Republic of China; 4 Research Institute of Tuina, Shanghai Academy of Traditional Chinese Medicine, Shanghai, 200437 People’s Republic of China

**Keywords:** Acupoint, Massage, Acupressure, Tuina, Mechanism

## Abstract

Acupoint massage is a non-invasive traditional therapy that has demonstrated reliable clinical outcomes in pain management, mental health relief, sleep disorder regulation, gastrointestinal treatment, and as an adjunct therapy for cancer. Its convenience and cost-effectiveness further enhance its appeal. However, the existing English literature lacks a systematic review that encompasses the various forms of acupoint massage. The acupoint massage forms adaptability is particularly notable when considering the diverse conditions, it addresses, as well as its applicability across different age groups and gender differences. Providing a comprehensive understanding, it is crucial to outline common practices and explore specific applications in key areas. The comprehensive understanding can create opportunities for effective collaboration between preclinical and clinical studies. Defining and categorizing different forms of acupoint massage is essential, alongside investigating the neural circuits involved in touch sensation. Future efforts should enhance collaboration with modern biology, facilitating the transition from empirical to evidence-based practice. This review summarizes forms, applications, and mechanisms of mainstream acupoint massage and provides insights for future research and applications, promoting deeper integration into healthcare.

## Introduction

Amidst the swift evolution of societal structures, individuals face mounting work-related and existential stress, resulting in a surge of sub-health conditions. Simultaneously, the diversification and heightened incidence of chronic diseases present formidable challenges, with their symptoms and drug side effects creating significant obstacles [1]. While pharmacotherapy remains a prevalent treatment modality, its associated healthcare expenses are substantial, and escalating costs have not correspondingly improved health outcomes or quality of life [2, 3]. Furthermore, widespread drug use and dependence risk medication abuse and addiction [4]. In addition, the challenges posed by medication or other expensive treatments are especially evident in conditions such as chronic pain, depression, and insomnia, which contribute significantly to years lived with disability (YLD) and place increasing financial strain on healthcare systems [5–8]. As drug prices rise and the global economy falters, complementary and alternative medicine (CAM), offering lower costs and greater convenience, has garnered heightened interest [9].

Acupoint massage, with its diverse therapeutic effects, has achieved notable clinical outcomes. Previous studies [10, 11] have shown positive attitudes and willingness towards acupoint massage among patients and healthcare professionals, indicating its potential for widespread clinical application [12]. The human body comprises numerous pressure points, known as acupoints, corresponding to various organs and systems [13]. Each acupoint exhibits distinct therapeutic effects, and applying pressure at different points elicits varying outcomes, contingent on the specific condition or painful body part. The core of acupoint massage involves activating these acupoints through pressure stimulus, leading to sensations such as soreness, numbness, and distention, collectively termed “De Qi” [14]. Stimulating specific acupoints elicits functional responses with therapeutic potential, particularly in chronic diseases, where it could alleviate symptoms, disabilities, and financial burdens on patients [15].

Despite extensive research on acupoint massage within CAM, the current English literature lacks a systematic review encompassing its diverse forms. The variability in its effectiveness for similar conditions warrants attention, especially in scenarios where certain medications or treatments are infeasible or prohibitively expensive. Ensuring patients are not exposed to ineffective therapies when acupoint massage offers potential is imperative. Therefore, defining markers or features that stratify patients most likely to benefit from acupoint massage therapies is crucial.

This review summarises the applications, efficacy, mechanisms, and functions of various acupoint massage forms in disease management, exploring future trends and potential. It aims to foster interaction between preclinical and postclinical studies, providing insights and directions for subsequent clinical applications and fundamental investigations.

## The forms, regions, and distinctions of acupoint massage

Acupoint massage is one of the CAM that has gained widespread acceptance and utilization worldwide due to its convenience, low cost, and demonstrated efficacy and potential in promoting health and treating illnesses. The practice of acupoint massage encompasses diverse methodologies, particularly prevalent in the East world. Western massage techniques, exemplified by Swedish massage, primarily emphasize the use of essential oils to directly manipulate muscle tissues through techniques such as effleurage, petrissage, friction, and tapotement. Swedish massage core concept differs significantly from the precise stimulation of specific acupoints in traditional acupoint massage. Thai massage, integrates various muscle manipulation techniques like stretching, extending, kneading, and pinching. While not fully overlapping with the traditional definition of acupoint massage, it also incorporates intricate point-pressure manipulations in multiple rows, allowing force to penetrate deeply into the muscle layers and achieve strong stimulation of specific acupoints. The point-pressure manipulations characteristic approach aligns with the core principle of acupoint massage [16]. Reflexology shares numerous similarities with acupoint therapy in terms of its origins, application scopes, and definitions, as both emphasize stimulating specific body regions to regulate physiological functions of corresponding areas or the entire body for therapeutic effects. Given this, the present study will focus on the core aspects of traditional acupoint massage, primarily exploring acupressure, Shiatsu, auricular acupressure, Therapeutic Thai Acupoint Massage, and foot reflexology.

### Acupressure: an ancient technique

Acupressure, an ancient Chinese technique dating back over 5000 years, is rooted in the meridians and acupoints theory. Traditional Chinese medicine (TCM) posits twelve primary meridians, eight extraordinary meridians, and 365 regular acupoints within the human body. These meridians serve as channels for qi and blood circulation, vital for life activities, while acupoints act as hubs for their infusion into viscera and meridians. This theory’s framework was first outlined in the “Yellow Emperor’s Internal Classic” and has since developed into a comprehensive system through medical practice, the above explanations are consolidated into meridians and acupoints theory. Acupressure is widely used in Chinese households for self-administration and has demonstrated therapeutic effects in emergencies, such as coma, pain, and asthma, as well as in treating internal conditions like nausea and vomiting [17]. Moreover, it forms the basis for various acupoint massages and Tuina manipulations, including digital point pressing, kneading, and rubbing, with proven efficacy in pain management, including cancer pain [18–20].

### Shiatsu: a Japanese therapy

Shiatsu, meaning “finger pressure” in Japanese, involves applying stable, sustained pressure to acupoints using fingers, hands, elbows, knees, and feet [21]. Developed in Japan, it retains TCM principles while incorporating Japanese cultural elements, evolving into a therapeutic approach with distinctive Japanese characteristics. Shiatsu is practised globally, particularly in Europe, with unique styles and theoretical foundations, showing therapeutic benefits in pain and sleep disorder management [22]. Emerging forms, such as Watsu employ warm water immersion at approximately 35 °C to generate hydrostatic pressure that modulates fluid distribution dynamics, metabolic processes, and respiratory mechanics, with gravitational force reduction minimizing joint loading, enhancing postural flexibility, and improving clinical outcomes in the management of muscular injuries, musculoskeletal pathologies, and stroke rehabilitation [23], are undergoing further development and study [24].

### Auricular acupressure: targeting the ears

Auricular acupressure is based on the concept that each body organ has a corresponding reflection point on the external ear. This method stimulates specific ear points for disease prevention, diagnosis, and treatment. With a 2000-year history in China, it evolved into a comprehensive system. In the 1950s, French medical doctor Nogier proposed 42 auricular points and an auricular map resembling an embryonic silhouette. Simultaneously, Chinese doctors integrated the meridians and acupoints theory of TCM, further enriching the fundamental principles of auricular acupressure and providing more precise operational guidelines. A standardized auricular therapy, integrating Chinese and Western systems, has emerged and is effectively applied in multiple countries, particularly demonstrating promising efficacy in treating chronic pain in the head and back regions [25].

### Therapeutic Thai acupressure: a deep approach

Therapeutic Thai acupressure involves applying pressure with the thumbs onto specific acupoints located along meridian lines, with the pressure tailored to each recipient’s pain threshold to ensure both comfort and efficacy. Multiple, repetitive pressing motions enhance therapeutic effects, while multi-layered and multi-angled massage techniques target acupoints, deeply penetrating muscles [26]. Popular in Southeast Asia, Thai massage is highly effective in treating chronic muscle pain due to its strong stimulation [27].

### Foot reflexology: stimulating the feet

Foot reflexology, believed to have originated in China over 5000 years ago, was adapted by physical therapist Eunice D. Ingham in 1930. She expanded its theoretical and practical foundations, forming the basis of modern reflexology [28]. This therapy involves applying pressure to specific areas of the feet, which possess reflex zones and points associated with body parts, organs, and glands. Accessible and sensitive reflex points allow for targeted pressure, eliciting responses in corresponding organs or glands, achieving therapeutic goals [29]. Popular in East Asia and the West, with studies in Central Asia and the Middle East, foot reflexology is particularly embraced by parturient women due to its efficacy in relieving labour pain and anxiety, without the costs and side effects of pharmacological interventions [30].

### Distinctions in acupoint massage

Acupoint massage techniques universally aim to stimulate specific regions for therapeutic benefits, either locally or systemically. Despite their shared goals, subtle variations among these forms exist, yet these differences may not markedly impact treatment efficacy, leading to comparable outcomes.

#### Acupressure versus Shiatsu

Firstly, Shiatsu tends towards holistic discipline, whereas acupressure places greater emphasis on targeting specific acupoints to precisely improve the function of related regions or organs. Secondly, the pressure applied in Shiatsu remains stationary and sustained, with both hands rhythmically pressing and moving along specific meridians. In acupressure, the pressure applied takes various forms, ranging from a pumping action involving repeatedly presses and releases pressure quickly to a vertical pressing that gradually increases in depth and intensity. Lastly, Shiatsu often utilizes both hands, with the thumbs consistently held in an extended position, and relies primarily on the practitioner’s body weight to apply pressure. This approach facilitates the stable application of pressure and ensures the longevity of the manipulation. In contrast, acupressure employs a more diverse range of manipulations, applying pressure through varying methods and utilizing differing degrees of force across different body parts based on the specific manipulation and need, thereby achieving a more precise and flexible treatment [21]. Although there are some small differences, but generally follow the meridians and acupoints theory and the perspective of the site of pain is Shu (acupoint).

#### Auricular acupressure versus foot reflexology

Both auricular points and foot reflexology zones are reflections from the perspective of biological holism, illustrating the intimate connection between parts and the whole of the human body, but they also exhibit distinctions. Firstly, auricular acupressure is relatively more convenient, with commonly used methods including the prolonged fixed pressure application of vaccaria seeds or magnetic pearls, which patients can administer themselves. In contrast, foot reflexology often necessitates the involvement of another person. While self-administered massage can be achieved with tools such as acupressure mats, the experience of receiving massage from another person imparts a heightened sense of pleasure and therapeutic potential, rendering its overall convenience and autonomy slightly inferior to auricular acupressure [31]. Secondly, the manipulations employed differ due to the distinct physiological structures involved. In auricular acupressure, the kneading method is a commonly utilized manipulation that can precisely target auricular points. In contrast, foot reflexology therapy often uses finger joints pointing method, which typically involves a greater force and intensity of stimulation compared to auricular acupressure.

**Fig. 1:**
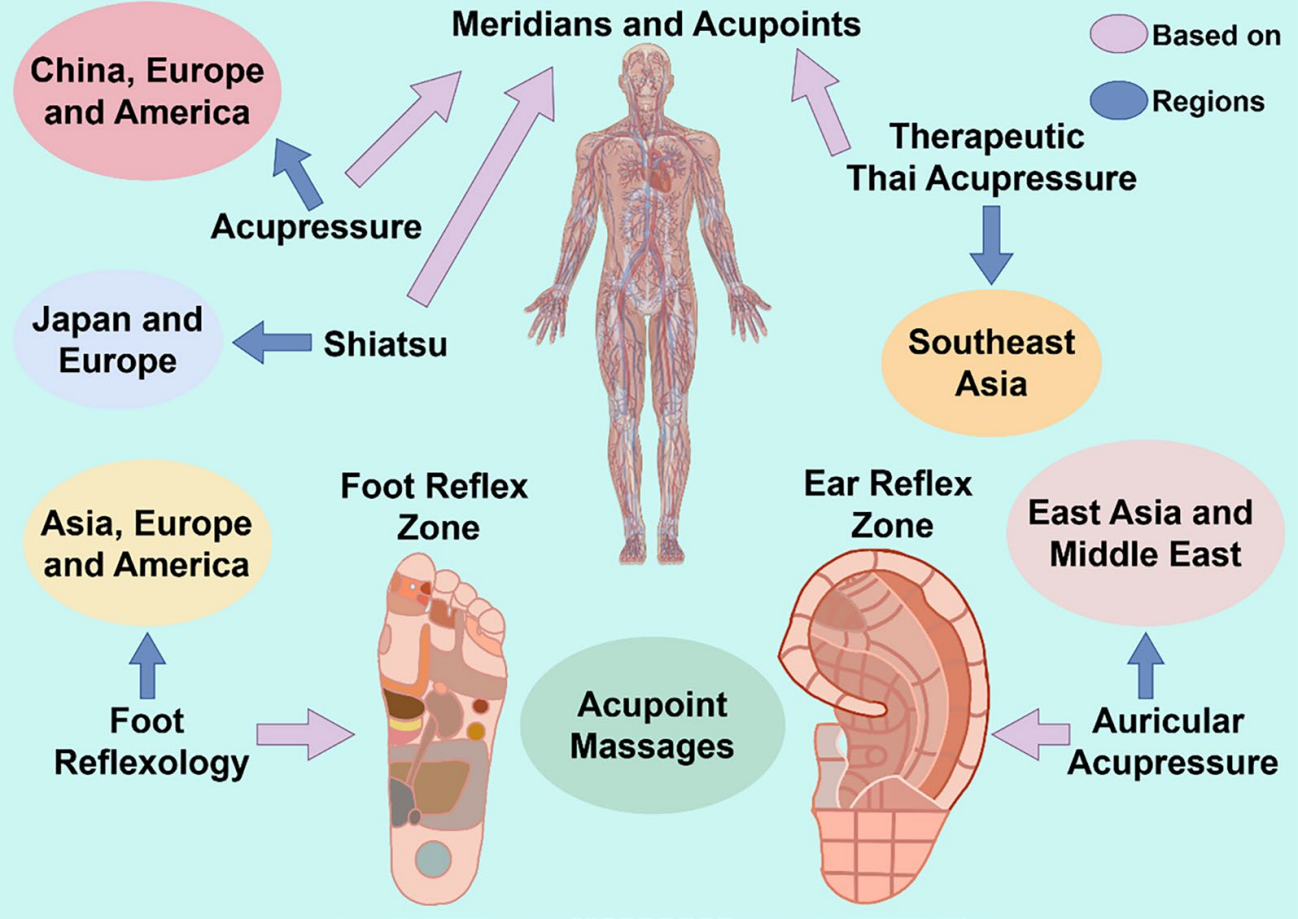
The four forms of region and theory. Acupressure is based on the theory of meridians and acupoints and is mainly used in China, Europe and the America; Shiatsu is based on the theory of meridians and acupoints and is mainly used in Japan and Europe; Therapeutic Thai Acupressure is based on the theory of meridians and acupoints and is mainly used in Southeast Asia; Foot Reflexology is based on the Foot Reflex Zone and is mainly used in Asia, Europe and America; Auricular Acupressure is based on the Ear Reflex Zone and mainly used in East Asia and Middle East

In summary, this part delves into the rich history, theoretical foundations, and diverse applications of acupressure and related therapies (Fig. 1) (Table 1). Their proven efficacy in treating various health conditions underscores their significant academic value and practical importance, warranting further research and integration into contemporary healthcare practices. Furthermore, it elucidates the nuanced distinctions between different acupoint massage techniques, highlighting their unique features while acknowledging their overarching therapeutic similarities. The therapeutic application of acupoint massage demonstrates notable regional variations rooted in their distinct cultural and geographical origins, resulting in the development of localized treatment traditions. As these techniques continue to undergo global dissemination and scientific investigation into their therapeutic mechanisms, development of international guidelines highlighting the benefits of specific techniques for different conditions and clinical applications progresses, the selection of acupoint massage forms is gradually evolving toward greater diversity and condition-specific adaptation. These insights deepen our understanding of acupoint massage methodologies, guiding practice and enhancing their potential for targeted, effective treatments.

**Table 1:** Forms, pressure intensity, primary acupoints and theoretical basis of acupoint massage

Form	Pressure intensity	Primary acupoints	Theoretical basis
Acupressure	Variable	Full-body acupoints	Meridians and acupoints theory
Shiatsu	Moderate to deep	Full-body acupoints	Meridians and acupoints theory
Auricular Acupressure	Light to moderate	Ear reflex zone	Ear reflex zone and TCM theory
Therapeutic Thai Acupressure	Deep	Full-body acupoints	Meridians and acupoints theory
Foot Reflexology	Light to deep	Foot reflex zone	Foot reflex zone and TCM theory

**Fig. 2:**
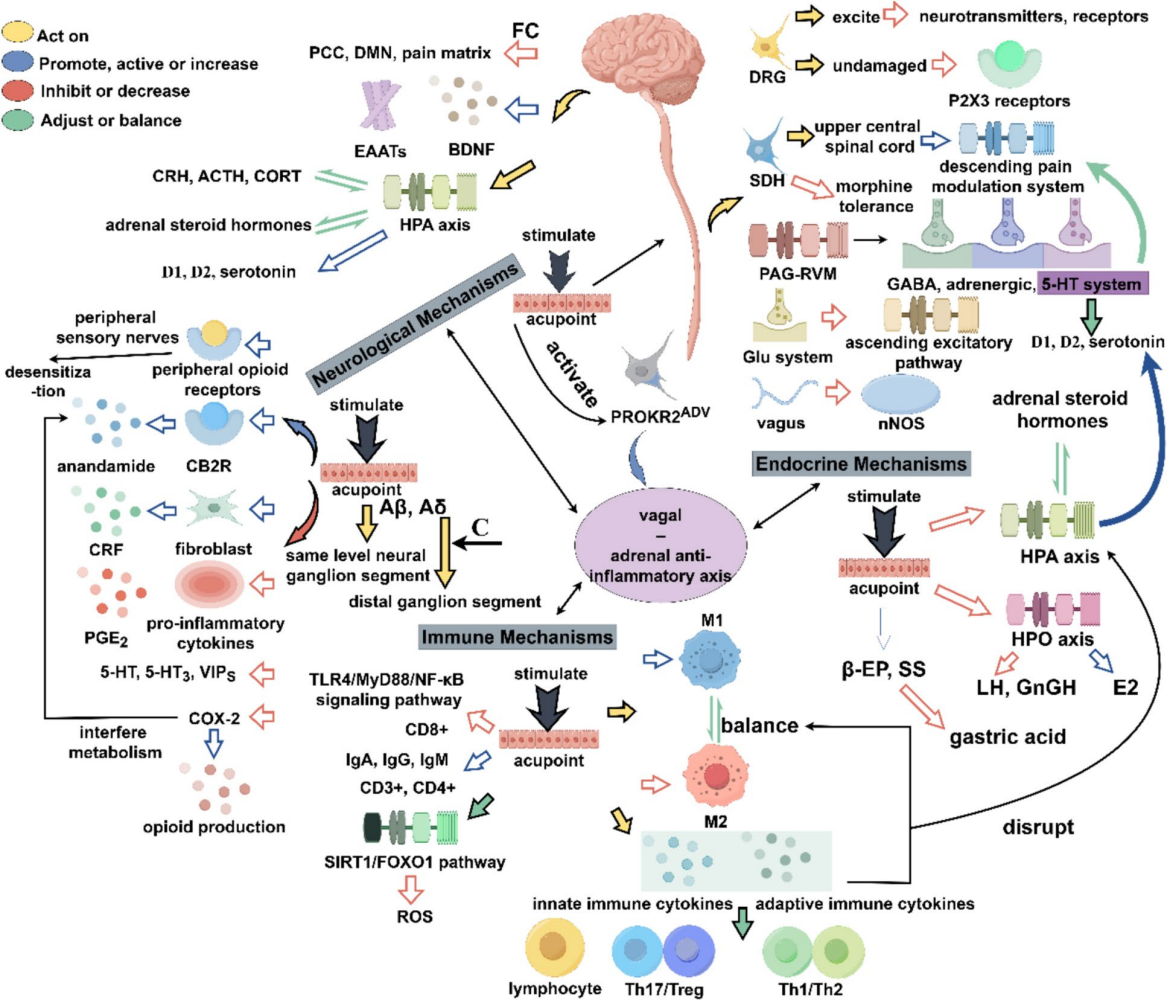
The main underlying mechanisms of acupoint massage. *PCC* posterior cingulate cortex, *DMN* default mode network, *FC* functional connectivity, *EAATs* excitatory amino acid transporter, *CRH* corticotropin-releasing hormone, *ACTH* adrenocorticotropic hormone, *CORT* cortisol, *HPA* hypothalamic–pituitary–adrenal, *D1* dopamine D1, *D2* dopamine D2, *CB2R* cannabinoid CB2 receptor, *CRF* corticotropin-releasing factor, *PGE*_*2*_ prostaglandin E_2_, *5-HT* 5-hydroxytryptamine, *5-HT*_*3*_ 5-hydroxytryptamine 3, *VIP*_*S*_ vasoactive intestinal peptides, *COX-2* cyclooxygenase-2, *ROS* reactive oxygen species, *M1, M1* macrophages, *M2, M2* macrophages, *DRG* dorsal root ganglia, *SDH* spinal dorsal horn, *PAG-RVM* periaqueductal gray-rostral ventral medulla, *GABA* gamma aminobutyric acid, *Glu* glutamate, *nNOS* nitric oxide synthase, *β-EP* beta-endorphin, *SS* somatostatin, *HPO* hypothalamic-pituitary-ovary, *E2* estradiol, *LH* luteinizing hormone, *GnRH* gonadotropin-releasing hormone

## Clinical applications of acupoint massage

Acupoint massage has transcended its traditional roots to emerge as a modern therapeutic modality with an array of clinically proven benefits. Its focus has shifted seamlessly from empirical practices to evidence-based treatments, tailored to specific disease profiles, patient demographics, and individual needs. This non-invasive therapeutic approach not only allows patients to manage their health effectively within the home environment but also offers a viable adjunctive treatment option for modern healthcare. As study into the efficacy of acupoint massage deepens and clinical evidence continues to accumulate, its prospects for application in contemporary medicine appear increasingly promising. This review systematically examines the therapeutic potential of acupoint massage across four major clinical domains: pain management, psychiatric conditions, sleep disturbances, and gastrointestinal disorders. By critically evaluating the current evidence regarding clinical applications, therapeutic outcomes, and methodological limitations of various acupressure techniques, this review aims to establish a comprehensive framework for exploring potential mechanisms, facilitating the translation of basic research findings into clinical practice while serving as a bridge between preclinical research and postclinical applications in the future.

### Pain relief: a holistic approach

Pain, an unpleasant sensory and emotional experience resulting from tissue damage, drives patients to seek medical attention [32]. Pain is categorised as transient, acute, or chronic, with acute and chronic pain management being of paramount importance. Acute pain interventions aim to alleviate discomfort and accelerate healing, whereas chronic pain is more intricate, often persisting and disrupting normal physiology. Temporary alleviation alone does not address underlying causes. Both chronic pain and severe depression significantly contribute to YLD, highlighting the urgency for effective and accessible pain management interventions [5, 6].

During labour, women experience intense pain exacerbated by physical tension, anxiety, and fear. Sanyinjiao (SP6), recognized as a key therapeutic target in obstetrical interventions, effectively mitigates labour pain through acupressure. Continuous acupressure on SP6 for 30 min has been shown to reduce visual analogue scale (VAS) scores, cesarean section rates, and shorten the second stage of labour, minimising oxytocin use [33, 34]. Additionally, pre-cesarean acupressure on SP6 and Hegu (LI4) acupoints alleviates post-cesarean pain and reduces analgesic consumption [35]. Foot reflexology demonstrates therapeutic efficacy in labour pain management, showing dual benefits in promoting maternal relaxation and reducing postpartum afterpain, preliminary evidence suggests its potential influence on lowering cortisol (CORT) levels and enhancing serotonin and dopamine activity [36, 37].

Acupoint massage demonstrates significant potential in alleviating pain symptoms in other gynaecological conditions. In cases of spasmodic, congestive, and primary dysmenorrhea, acupressure at SP6 and LI4 demonstrates improvements in pain intensity, with analgesic effects persisting beyond 2 h post-intervention [38, 39]. Auricular acupressure targeting specific ear points, such as genital, endocrine, and Shenmen, provides non-pharmacological relief for primary dysmenorrhea, reducing VAS scores and symptom severity following intervention. While the temporal onset of dysmenorrhea episodes may exhibit variability, the accessibility of relevant acupoints permits self-administered stimulation across diverse settings, enabling timely symptom mitigation. Moreover, auricular acupressure and foot reflexology have also demonstrated good therapeutic effects for managing procedure-related abortion pain and lactation-associated breast discomfort [40, 41].

In chronic pain management, acupressure exhibits notable efficacy. A systematic review encompassing 486 patients with chronic low back pain revealed that acupressure interventions were associated with statistically significant reductions in pain severity and sustained analgesic outcomes [42]. Targeting specific auricular acupoints, such as liver, kidney, lumbar vertebrae, and Shenmen, provides immediate relief and increases the pain threshold [43, 44]. Shiatsu effectively in managing neck, back, and lower back pain, while economic evaluations suggest potential cost–benefit advantages in angina pectoris care protocols [45, 46]. Therapeutic Thai acupressure, a deep tissue massage, demonstrates therapeutic potential for chronic pain and fatigue management in soft tissue pathologies. Clinical trial data indicate symptom reduction in chronic lumbar region pain and myofascial pain syndrome, with preliminary evidence suggesting concomitant alleviation of comorbid anxiety states associated with these conditions [27, 47, 48]. For knee osteoarthritis, acupressure mitigates symptoms like swelling, pain, and joint dysfunction, improving joint function and reducing non-steroidal anti-inflammatory drugs usage. Its high incremental cost-effectiveness ratio underscores acupressure’s potential to reduce treatment costs while maintaining safety and tolerability [15, 49, 50]. Moreover, self-administered acupoint massage at home enhances convenience and practicality [51, 52].

### Mental wellness: a soothing power

Anxiety, a normal stress response, can escalate into physical symptoms such as increased heart rate, insomnia, and gastrointestinal distress. Severe cases may manifest as restlessness, tremors, muscle tension, and breathlessness. Depression, a prevalent mental disorder influenced by social, psychological, and biological factors [53], can lead to symptoms including anxiety, behavioural disorders, and weight disturbances. Traditional treatments like psychotherapy and medication face challenges: therapist shortages, high costs, and potential side effects or addiction risks from long-term medication use [54, 55]. Physical therapies, such as electroconvulsive therapy and transcranial magnetic stimulation, may induce seizures [56]. Acupoint massage, a non-invasive therapy, has shown therapeutic effects on mental disorders with low cost and high safety.

Post-cesarean section, women often exhibit increased susceptibility to acute postoperative pain, anxiety, and fatigue, factors that may collectively impair postpartum functional recovery. Compared to vaginal deliveries, cesarean sections as an independent risk factor for heightened incidence of postpartum depressive and anxiety symptoms with systematic analysis reporting [57, 58]. Drug therapy limitations for breastfeeding women highlight non-invasive therapies’ advantages. Auricular acupressure, including Shenmen, endocrine, subcortex, and uterus points administered at 5-day intervals, demonstrate statistically significant reductions in validated postpartum depression rating scales, resting heart rate parameters, and serum cortisol concentrations [59, 60]. Foot reflexology also shines in post-surgical recovery, reducing anxiety scores after just 20 min [61]. Preoperative anxiety, affecting over 90% of adults, can elevate blood pressure, heart rate, and anaesthetic requirements, increasing surgical risks [62]. Acupressure demonstrates significant efficacy in mitigating preoperative anxiety, stabilizing heart rate, and reducing CORT levels during elective surgical procedures, particularly evident when acupressure protocols are implemented in abdominoperineal, gynaecological, orthopedic, cardiovascular, plastic, and cesarean surgeries [63].

Non-surgical anxiety, depression, and other mental disorders are more prevalent among patients. Multiple systematic reviews and meta-analyses have revealed the effectiveness and broad patient acceptance of acupoint massage [14, 64, 65]. Foot reflexology boosts oxytocin release in brain regions associated with social cognition and reward processing, suggesting potential therapeutic applications for autism spectrum disorder symptom management [31]. Shiatsu reduces agitation in mechanically ventilated patients, enhancing treatment strategies when combined with pharmacotherapy [66]. In Alzheimer’s patients, Shiatsu plus physical activity decreases depression and improves cognitive function and functional status [67]. Academic populations frequently experience heightened stress responses during examination periods, with chronic academic pressure correlating with impaired cognitive performance and increased risk of stress-related somatic manifestations. Auricular acupressure, targeting Shenmen, endocrine, and cortical points, effectively reduces moderate to severe test anxiety, allowing students to maintain practical feasibility for implementation within academic settings due to its non-disruptive administration protocol and minimal time investment requirements [68, 69].

### Restorative sleep: a gateway to rejuvenation

Insomnia, a widespread sleep disorder marked by difficulties in initiating, maintaining, and re-initiating sleep, as well as early morning awakening, affects over half the global population. This condition often coexists with other mental and sleep disorders, such as restless legs syndrome (RLS) [70]. In the United States, the rising incidence of insomnia has led to substantial economic losses and increased healthcare costs [7, 8]. Cognitive behavioural therapy for insomnia is the recommended first-line treatment, with medications serving as a secondary option. However, challenges persist, including high costs and a shortage of qualified professionals [70, 71].

Acupoint massage has emerged as a convenient, cost-effective, and safer alternative to acupuncture for self-management of insomnia [72]. Clinical evidence increasingly supports acupoint massage as an effective treatment for insomnia, producing significant improvements in validated sleep assessment metrics, significantly reducing scores on the Pittsburgh sleep quality index (PSQI) and insomnia severity index. Acupressure has shown therapeutic effects in sleep onset latency and increases in total sleep duration. The acupoint Shenmen (HT7) is frequently incorporated into therapeutic protocols and is commonly combined with other acupoints, such as Yongquan (KI1) and Taixi (KI3), to address specific symptom profiles [73, 74]. Despite these advances, current evidence indicates a need for additional high-quality clinical trials to investigate the therapeutic efficacy of alternative acupoint combinations in insomnia management, as the existing literature demonstrates limited exploration of non-primary acupoint protocols. Targeted application of magnetic pearls to key auricular acupoints including Shenmen, endocrine, and heart regions demonstrates clinically have been found to extend sleep duration and improve sleep quality. Clinical trial data indicate Shiatsu interventions demonstrate measurable improvements across multiple sleep quality parameters in chronic pain and post-concussion populations, including statistically significant reductions in sleep onset latency and increases in total sleep duration. Concurrent improvements in fatigue-related daytime functioning measures have also been documented [75, 76]. Optimizing sleep structure through Shiatsu may provide valuable guidance for exploring larger sample sizes or more diverse sleep issues related to various diseases. Foot reflexology, another modality, demonstrates considerable versatility in acupoint selection protocols while maintaining clinically relevant efficacy in sleep regulation. RCTs document measurable improvements in sleep onset latency reduction and sleep efficiency metrics, positioning this modality as a viable component within multimodal insomnia management strategies [51, 77].

In hemodialysis patients, continuous acupressure on auricular acupoints for 8–12 weeks reduced sedative use and improved sleep duration and quality, and lower PSQI scores [78]. However, a systematic analysis of 618 hemodialysis patients receiving auricular acupressure therapy indicates that existing clinical studies confirms these preliminary findings while identifying critical methodological limitations, particularly insufficient sample sizes and protocol standardization, that necessitate validation through multicenter randomized controlled trials with extended follow-up periods [79]. RLS represents a prevalent neurological comorbidity in hemodialysis patients, demonstrating significant associations with impaired sleep architecture and elevated the risk of cardiovascular diseases and mortality [80, 81]. A pilot study administering acupressure three times weekly during hemodialysis showed potential in reducing RLS severity metrics, however, sleep quality did not significantly improve [82]. Therefore, while existing evidence suggests therapeutic potential for acupoint stimulation in managing RLS-related sleep disturbances within this population, methodological limitations in current trial designs, particularly regarding acupoint selection protocols and treatment duration optimization, necessitate rigorous multicenter RCTs to establish evidence-based clinical guidelines. These clinical evidence and limitations inform the refinement of therapeutic protocols and improvement of patient-centered outcomes in sleep disorder management, highlighting the necessity for methodologically rigorous research to elucidate the mechanisms underlying acupoint massage interventions and establish standardized treatment parameters across diverse clinical populations.

### Gastrointestinal harmony: nourishing from within

Constipation, marked by reduced bowel frequency and difficult defecation, is a prevalent gastrointestinal disorder often accompanied by bloating and abdominal pain [83]. Nausea and vomiting also frequently occur, sometimes chronically. Pharmacotherapy, while traditionally the mainstay of treatment, often proves inadequate and carries safety concerns for long-term use. Recent therapeutic approaches, such as physical activity and dietary adjustments, show promise but rely heavily on patient compliance, which can be challenging to achieve [84, 85]. Thus, there remains a pressing need for more effective, safe, and patient-adherent interventions.

During pregnancy, gastrointestinal issues are particularly common, with nausea and vomiting affecting up to 70% of women and constipation impacting over 40% [86]. Severe cases can lead to complications such as dehydration and electrolyte imbalances. Constipation, specifically, may weaken pelvic floor muscles, increasing the risk of uterovaginal prolapse [87–89]. Given the economic burden and side effects of drugs, non-pharmacological therapies are preferred. Acupoint massage, notably, shows therapeutic potential [90]. Neiguan (PC6) is a key acupoint in nausea and vomiting management, with clinical studies demonstrating therapeutic utility in obstetric populations. In a study involving 1378 pregnant women and a meta-analysis of 3,390, 90% used PC6 acupressure, which effectively reduced symptoms severity, decreased hospitalizations, and improved quality of life [91, 92]. Additionally, self-administered acupressure on Zhigou (TE6), when performed twice daily for 15-min sessions over a week significantly decreased constipation severity, promoting body fluid re-secretion and increasing blood volume [93]. Acupoint massage demonstrates consistent therapeutic efficacy across populations with varying comorbid obstetric conditions, underscoring its clinical utility in managing pregnancy-associated gastrointestinal dysfunction [94].

Beyond pregnancy, acupoint massage has also proven effective for managing nausea, vomiting, and constipation across diverse clinical contexts. Observational data from pediatric and postoperative cohorts further indicate a favorable safety profile with well-tolerated intervention protocols [95]. In stroke patients, acupressure on Tianshu (ST25) and Zhongwan (CV12) points reduced the need for constipation medication and improved bowel conditions [96]. Applying metallic beads to specific auricular acupoints increased spontaneous bowel movements and improved stool consistency [97]. While foot reflexology has shown therapeutic effects for constipation, though current evidence remains insufficient to establish its preventive effects on symptom recurrence or comorbid gastrointestinal manifestations [98]. Acupoint massage emerges as a viable therapeutic option with demonstrated tolerability and patient adherence profiles, providing a non-pharmacological intervention strategy for functional gastrointestinal pathologies.

### Beyond the ordinary: expanding the horizons of therapy

Acupoint massage has demonstrated remarkable efficacy in treating various diseases and offers promising potential for managing additional health conditions. In a study of pregnant women at 32–35 weeks of gestation, daily bilateral Zhiyin (BL67) acupressure for 2 weeks, each session lasting 10 min, facilitated spontaneous fetal rotation from breech to cephalic presentation. This intervention reduced the cesarean section rate and prevented complications associated with breech deliveries, providing a non-invasive, non-pharmacological alternative [99]. In pediatric populations with obesity, auricular acupressure interventions administered over an 8-week period demonstrate measurable reductions in anthropometric parameters, specifically waist and hip circumference measurements, while maintaining BMI stability. These findings suggest a potential role for this non-invasive modality in adjunctive pediatric weight management strategies [100]. Therapeutic Thai acupoint massage, targeting specific acupoints along meridian lines, improved gait patterns, proprioceptive function, reduced visual dependency, and strengthened upper limb muscle power in Parkinson’s patients [26, 101].

Cancer, a global health burden with 20 million new cases and nearly 9.7 million deaths in 2022, poses significant societal and macroeconomic challenges [102]. Cancer treatment often results in excruciating pain and various complications, including nausea, vomiting, constipation, fatigue, and sleep disorders. Acupoint massage demonstrates potential as a non-pharmacological adjunct therapy for managing these symptoms, enhancing patients’ quality of life. Clinical analyses have revealed a significant correlation between acupoint massage and reduced pain intensity in cancer patients. Furthermore, it has shown promise in decreasing opioid-based painkiller usage, addressing the opioid abuse crisis [20, 103]. PC6 acupressure significantly reduced the frequency and severity of acute and delayed nausea and vomiting in chemotherapy patients [104]. However, no improvement was observed in pediatric patients undergoing highly emetogenic chemotherapy [105]. Auricular acupoints, such as heart, liver, Shenmen, and sympathetic points, demonstrated a significant inhibitory effect on acute nausea in breast cancer patients undergoing chemotherapy, with potential for managing acute vomiting [106]. Acupressure on CV12 and ST25 provided short-term relief of constipation symptoms in terminal cancer patients [107]. Auricular acupressure related to the rectum, colon, lungs, and subcortex improved constipation symptoms and quality of life in leukemia patients. Additionally, acupressure on Shenmen, sympathetic, heart, and subcortex acupoints significantly improved sleep scores and reduced sleep disorders and medication usage in lung cancer patients [108]. Foot reflexology produce measurable fatigue reduction in gastrointestinal oncology populations during the 24-h post-chemotherapy observation window [109], however, did not significantly improve other ancillary symptom clusters among palliative care cohorts [110]. But it’s still worth noting, the above parts of virous other diseases can be seen that different forms of acupoint massage still have considerable effects on many types of miscellaneous diseases.

Acupoint massage offers a promising alternative to traditional therapies for managing pain, mental disorders, sleep disturbances, gastrointestinal issues, and various other health conditions. Its non-invasive, cost-effective, and safe nature, coupled with the ability to tailor treatments to individual needs, underscores its potential to revolutionize modern healthcare. As research continues to uncover its therapeutic benefits and optimal acupoint selection, acupoint massage stands poised to make significant contributions to improving patient outcomes and enhancing quality of life. The main regions and advantageous diseases are summarised in Table 2.

**Table 2:** Forms, main regions and advantageous diseases of acupoint massage

Forms	Main regions	Advantageous diseases
Acupressure	China, Europe and America	Pain
		Mental disorders: depression, anxious
		Sleep disorders
		Gastrointestinal diseases: constipation nausea and vomiting
		Cancer-related symptoms
Shiatsu	Japan and Europe	Pain
		Mental disorders: depression anxious AD agitation
		Sleep disorders
Auricular acupressure	East Asia and the Middle East	Pain
		Mental disorders: depression anxious
		Sleep disorders
		Gastrointestinal diseases: constipation
		Cancer-related symptoms
		Obesity
Therapeutic Thai acupressure	Southeast Asia	Pain
		PD
Foot reflexology	Asia, Europe and America	Pain
		Mental disorders: autism
		Sleep disorders
		Gastrointestinal diseases: constipation

Massive meta-analyses of RCTs report statistically significant outcomes, such as reduced VAS and PSQI scores, yet some key limitations warrant consideration. First, methodological heterogeneity persists across studies, with variability observed in acupoint selection protocols, such as differing applications of SP6 and HT7 for insomnia, and intervention parameters, including treatment duration and session frequency. This variability introduces challenges for cross-study comparisons, as seen in nonsignificant outcomes for chemotherapy-induced nausea and vomiting in pediatric populations, where variations in technique application and cohort characteristics may contribute to divergent results. What’s more, methodological constraints include instances of suboptimal blinding implementation and modest sample sizes in a proportion of RCTs, factors that could potentially influence effect size estimations. Additionally, Publication bias may further skew the evidence base, as negative results are underrepresented. The cumulative evidence nevertheless supports acupoint massage as a feasible adjunctive therapy. To strengthen clinical guidance, future research would benefit from prioritizing multicenter RCTs employing standardized protocols with enhanced blinding strategies and longitudinal follow-up. Such efforts could improve comparability across studies, refine efficacy validation, and clarify optimal therapeutic parameters for specific patient subgroups.

## The possible mechanisms of acupoint massage

The mechanism of acupoint massage may have many explanations, and its deep physiological effect mechanism shows a high degree of complexity and interaction. The stimulation triggered by acupressure may first be captured by the neural network and quickly respond, triggering a wide range of nerve pathways. This initial neuro-mediated response then triggers a series of immune and endocrine system regulations. However, the physiological effects of acupoint massage don’t exist in isolation, but constitute an interacting neuro-endocrine-immune regulatory network (Fig. 2).

### Neurological mechanisms

#### Peripheral mechanisms

Acupoint massage entails applying pressure to specific points, eliciting the sensations such as soreness, numbness, and distention termed “De Qi”. Stimulation of acupoints, coupled with electromyography (EMG) recordings, reveals a positive correlation between EMG wave amplitudes, subjective stimulation intensity, and “De Qi” sensation. However, anaesthesia of muscles or lumbar vertebrae associated with these acupoints abolishes both EMG signals and “De Qi” [111]. Rodent studies indicate a higher density of mast cells near the ST-36 acupoint compared to adjacent non-acupoint areas. Disrupting mast cells pharmacologically attenuates analgesic effects [112], yet collagenase injection at acupoints does not necessarily negate visceral pain inhibition, whereas local anaesthesia maintains pain-blocking properties [113]. Above phenomenon suggests peripheral nerve tissue, rather than connective tissue, is pivotal in acupoint activation. Nonetheless, mast cells are not the sole initiators of analgesia. This is evidenced by rats lacking mast cells, which exhibit attenuated mechanical, but not thermal, analgesia [114].

Regarding afferent nerves, four fibre types exist: Aα (Type I), Aβ (Type II), Aδ (Type III), and C (Type IV). Aβ, Aδ, and C fibres are implicated in acupoint analgesia, with ongoing debate on their respective roles [115, 116]. Acupoints are rich in various receptors that respond to stimuli such as pain, itching, temperature, mechanical forces, and tissue damage. The mechanical force applied during acupoint stimulation is sensed by mesenchymal stem cells in the acupoint region, which initiate a mechanical transduction process through corresponding ionic changes and intracellular signaling. The sensation of “De Qi” may be mediated by multiple afferent nerve fibers simultaneously, leading to a mixed sensory experience, including soreness, numbness, and distention [117]. Stimulation of deep acupoint areas activates Type II and a few Type III fibres, achieving significant analgesia at the same neural ganglion segment. For distal segments, C fibre involvement is crucial for acupoint analgesia. Aδ and C fibres primarily mediate visceral pain, hence activating corresponding Type III or IV fibres at specific acupoints induces analgesia. In the special microenvironment at the acupoint induced by stimulation, local connective tissue deformation leads to the release of various biochemical mediators, including interleukin, tumor necrosis factor, and adenosine triphosphate (ATP). ATP activates afferent fibers via P2X receptors, and when ATP is broken down into adenosine, it can activate adenosine A1 receptors on C and Aδ fibers, contributing to analgesic effects. Acid-sensing ion channel 3 (ASIC3) and transient receptor potential (TRP) channels are present in mechanical ion channels. ASIC3 mainly mediates acid and mechanical reactivity in Aβ fibers, which are predominant in skin and muscle, while TRPV1 channels are highly expressed in sensory Aδ and C fibers. The analgesic effect of low-intensity acupoint stimulation is mediated by ASIC3 receptors on Aβ fibers, while the systemic analgesic effect of high-intensity acupoint stimulation is mediated by Aδ and C fibers via TRPV1 receptors [118].

#### Central mechanisms

Stimulation of the acupoint ST36 enhances depolarization of primary afferent C-fibers at peripheral terminals, leading to inhibitory post-synaptic potentials in nociceptive neurons and sustained membrane hyperpolarization. This mechanism suggests a pivotal role for post-synaptic inhibition in the analgesic effects of acupoint massage [116]. The spinal cord serves as a central hub for modulating both ascending and descending pain signals, with dorsal root ganglia (DRG) acting as the first-order neurons. P2X3 receptors are implicated in neuropathic pain transmission, and acupoint stimulation may inhibit DRG neuron excitation and associated neurotransmitter expression, alleviating pain and potentially reversing P2X3 receptor upregulation, which contributes to pain hypersensitivity. However, the regulation phenomenon of the DRG mentioned above effect may be limited by various chronic pain models [119, 120]. Furthermore, it downregulates transient receptor potential vanilloid subfamily 1 mRNA and protein expression in the spinal dorsal horn (SDH), further contributing to pain relief [121]. Acupoint stimulation activates SDH neurons, transmitting impulses to the upper spinal cord and engaging the descending pain modulation system. The acupoint stimulation activation exerts multimodal analgesic effects through SDH receptor modulation targeting 5-hydroxytryptamine (5-HT) and *N*-methyl-d-aspartate receptor 1 systems, concurrent β-endorphin expression upregulation, and suppression of algogenic neurotransmitter release pathways [122, 123]. Additionally, the activation downregulates key signalling proteins, *p*-phosphoInositide-3 kinase and p-protein kinase B, within the SDH, delaying morphine tolerance and enhancing analgesic efficacy [124].

Brain structures, including the primary and secondary somatosensory cortex, anterior cingulate cortex, amygdala, hippocampus, thalamus, and insula, are involved in analgesia regulation. Acupoint stimulation activates these regions, modulating limbic systems in pain areas, balancing central homeostasis, and contributing to the regulation of the descending pain system [125]. It also reduces functional connectivity among the posterior cingulate cortex, the default mode network, and most regions of the pain matrix [126]. Moreover, acupoint stimulation alters the activity of brain nuclei associated with gastrointestinal function, regulating gastrointestinal motility through integrated analysis via the autonomic nervous system, intrinsic nervous system, and glial cells [127]. Stimulation of lumbar and hindlimb segments modulates dopamine and serotonin levels in various brain regions, addressing gastrointestinal symptoms and possessing anti-stress functions [128, 129].

Abdominal acupoint stimulation primarily activates sympathetic nerve efferent fibers, whereas limb acupoint stimulation inhibits gastrointestinal motility through vagal nerve efferent fiber activation [130, 131]. Stimulation of ST25 downregulates nitric oxide synthase (nNOS) expression, while Shangjuxu (ST37) exerts dual regulatory effects, downregulates nNOS and upregulates choline acetyltransferase levels, restoring enteric neural homeostasis and ameliorating gastrointestinal dysmotility [132]. Furthermore, ST36 stimulation activates enteric glial cells, secreting substances that regulate intestinal function via cholinergic anti-inflammatory pathways, thereby reducing intestinal damage, preventing intra-abdominal adhesions, and decreasing the risk of intestinal obstruction [133, 134].

### Endocrine mechanisms

Acupoint stimulation exhibits a profound impact on the regulation of the hypothalamic–pituitary–adrenal (HPA) axis. By inhibiting overactivity within the axis, it reduces levels of key hormones such as corticotropin-releasing hormone (CRH), adrenocorticotropic hormone (ACTH), CORT, and orexin-A. Concurrently, it enhances the expression of dopamine D1 and D2 receptors and serotonin within the HPA axis, thereby improving sleep quality [135, 136]. This acupoint stimulation also elevates melatonin levels in the pineal gland, suprachiasmatic nucleus, and ventrolateral preoptic nucleus, leading to increased mRNA expression of melatonin receptors MT1 and MT2. Furthermore, it amplifies gamma aminobutyric acid (GABA) and dopamine transmission within the striatum, reducing sleep onset latency and extending total sleep duration. Additionally, acupoint stimulation modulates the dysregulation of Bmal1 and Per2 mRNA expression associated with insomnia, normalises brain-derived neurotrophic factor (BDNF) level fluctuations, and mitigates endoplasmic reticulum stress in the medial septum of rats [137–139]. Moreover, it inhibits the hypothalamic-pituitary-ovary (HPO) axis, decreasing estradiol levels while increasing luteinizing hormone and gonadotropin-releasing hormone, all of which contribute to coordinated endocrine modifications exhibit regulatory influences on sleep physiology [140, 141]. It is noteworthy that the stimulation of acupoints can elicit a distinct physiological response. Specifically, the stimulation of acupoints increases the production of beta-endorphin and somatostatin, which in turn suppress gastric acid secretion and alleviate symptoms associated with gastrointestinal mucosal lesions [142]. Thus, the stimulation of these different acupoints offer a holistic approach to managing various health conditions.

Furthermore, acupoint stimulation affects energy metabolism and hormonal functions by decreasing levels of leptin, growth hormone releasing peptide, insulin, and miR-32-3p, while upregulating group IV phospholipases A2 and stimulating skeletal muscle Sirtuin 1 (SIRT1)/peroxisome proliferator-activated receptor γ coactivator 1α. This intervention reverses insulin resistance [143–145]. Simultaneously, it elevates systemic estrogen and testosterone levels, promotes the synthesis and secretion of adrenal steroid hormones, and increases levels of integrin v3, homeobox A10, heparin-binding EGF-like growth factor, estrogen receptor alpha, and progesterone receptor. These effects, in turn, regulate HPA axis dysfunction and reduce physiological disturbances caused by sex hormone deficiency [146–148].

### Immune mechanisms

Stimulation of specific acupoints exhibits profound effects on modulating inflammatory responses and immune function. Stimulation of the Shenmen, subcortical, and sympathetic auricular points downregulates proinflammatory cytokines interleukin-1 beta (IL-1β), interleukin-2 (IL-2), and interleukin-6 (IL-6), while upregulating the anti-inflammatory cytokine interleukin-4 (IL-4) and decreasing calcitonin gene-related peptide. This mechanism alleviates inflammatory responses in chronic low back pain [149]. Furthermore, acupoint stimulation enhances levels of tight junction proteins including zonula occludens-1 (ZO-1), occludin, and claudin-5 in the damaged intestinal barrier during chemotherapy, suppressing inflammatory cytokines, which including tumor necrosis factor-α (TNF-α), IL-1β and IL-6 both locally and systemically [150].

Stimulation of ST36 regulates macrophage balance by inhibiting nuclear factor kappa-light-chain-enhancer of activated B cells (NF-κB) activation in pro-inflammatory M1 macrophages and promoting anti-inflammatory M2 macrophages, thus modulating the expression of cytokines and immune cell communication [151, 152]. Additionally, it stimulates both innate and adaptive immune cytokines, regulating lymphocyte balance and inhibiting inflammatory responses [153]. In colitis models, ST36 stimulation inhibits NLR Family, pyrin domain containing protein 3 and caspase-1 activation, decreasing IL-1β levels and reversing macrophage subset imbalances [151]. It also maintains immune system homeostasis by regulating Th17/Treg and Th1/Th2 cell balances [154]. Stimulation of PC6 significantly increases immunoglobulin levels and regulates T lymphocyte subpopulations, promoting immunoglobulin synthesis and secretion [155].

Furthermore, acupoint stimulation regulates the silent information regulator 1/forkhead box protein O1 pathway, inhibiting reactive oxygen species (ROS) -induced oxidative stress and reducing the expression of NOXs, ROS, and pro-inflammatory markers, thereby delaying cellular aging and exerting anti-inflammatory analgesic effects [156–158]. It also targets mitogen-activated protein kinase kinase kinase kinase 4 via microRNA-547-3p, inhibiting the NF-κB pathway and reducing local inflammatory factors in the DRG, improving the inflammatory microenvironment [159]. It is noteworthy that acupoint stimulation exerts additional impacts on inflammatory processes. Specifically, by inhibiting the Toll-like receptor 4/myeloid differentiation factor 88/NF-κB signalling pathway, acupoint stimulation diminishes the levels of pivotal inflammatory mediators while augmenting anti-inflammatory cytokines. This concerted action results in a reduced overall inflammatory response [160, 161]. These findings highlight the innovative potential of acupoint stimulation in modulating inflammatory and immune responses, with significant implications for the treatment of various inflammatory conditions and the enhancement of immune function.

### The neuro-endocrine-immune network

Acupoint stimulation can decrease the concentration of leptin in peripheral blood, inhibit the phosphorylation levels of AMP-activated protein kinase, increase mitochondrial membrane potential, and slow down mitochondrial damage [155]. In addition, acupoint stimulation can downregulate various pro-inflammatory cytokines, reducing the signaling from the peripheral nervous system to the central system, thereby inhibiting the excessive activation of brain regions such as the hypothalamus, and improving fatigue symptoms in cancer patients after chemotherapy [162].

Stimulation of specific acupoints, upregulates galanin and BDNF expression in the hippocampus, promoting nerve regeneration. It also modulates ACTH and CORT levels, inhibits high miR-16 expression, and improves depressive symptoms in model rats. Repeated stimulation significantly increases mature BDNF and proBDNF expression, promoting nerve regeneration in Alzheimer’s patients [163–165]. This approach not only addresses Alzheimer’s and Parkinson’s symptoms but also alleviates accompanying depression [166].

Additionally, acupoint stimulation can decrease the sensitivity of visceral nerves by downregulating the levels of peripheral chemical substances such as 5-HT, 5-HT_3,_ and vasoactive intestinal peptides, as well as decreasing the expression of their receptors, thereby relieving visceral pain [167–169]. The long-term analgesic effect of acupoint stimulation can also desensitize peripheral sensory nerves by activating peripheral opioid receptors, inhibiting immune cells release pro-inflammatory cytokines and the activation of PGE_2_, increasing the expression of the endogenous cannabinoid CB2 receptors, elevating anandamide levels, inducing fibroblasts to release corticotropin-releasing factor, and mediating crosstalk between the opioid and cannabinoid systems. Additionally, it can lower the concentration of cyclooxygenase-2, thereby interfering with the metabolism of endogenous cannabinoids, increasing opioid production levels, and achieving analgesic effects [170–172].

Glutamate (Glu) is a critical mediator of excitatory transmission in the central nervous system, with increased Glu concentration leading to pain signal transmission [173]. Acupoint stimulation modulates Glu and its receptors, inhibiting the ascending excitatory pathway by downregulating *N*-methyl-d-aspartate receptor type 2B and preventing α-amino-3-hydroxy-5-methyl-4-isoxazole-propionicacid receptor phosphorylation, thus promoting the recovery of glutamate transporters to clear excessive Glu, thereby contributing to analgesic effects [174, 175].

The descending pain modulation system is integral to the transition from acute to chronic pain. When acute pain transitions to chronic pain, the descending pathways take on a dominant role, leading to increased transmission of nociceptive signals and enhanced pain sensitivity. Acupoint stimulation modulates the descending pain modulation system by regulating the periaqueductal gray-rostral ventral medulla (RVM) pathway and associated neurotransmitters [176]. It activates GABA receptors and reduces GABA reuptake, achieving analgesic effects [177]. Additionally, stimulation activates spinal α2 and β-adrenoceptors, enhancing the anti-nociceptive response [178]. 5-HT levels, influenced by the RVM and spinal cord, are closely associated with analgesia. Acupoint stimulation activates 5-HT1 A receptors, alleviating pain in chemotherapy-induced neuropathy models and regulating the brain-gut axis [179, 180].

5-HT and Glu are also crucial in psychiatric disorder treatment. Acupoint stimulation in rats significantly enhances tryptophan hydroxylase and 5-HT1 A protein and mRNA expression in the hippocampus, increasing 5-HT synthesis and improving depressive behavior [181]. The dysregulated Glu system is closely associated with psychiatric disorders such as Alzheimer’s, schizophrenia, and anxiety. Acupoint stimulation can regulate Glu and its receptor expression, improving symptoms of these diseases [182]. It also enhances depressive states by increasing excitatory amino acid transporter 2 (EAAT2) positive cells and EAAT2 mRNA expression in the prefrontal cortex and hippocampus, alleviating depressive states through increased endorphin secretion and vagal nerve activation [59, 183, 184]. Pressing the Shenmen acupoint can enhance parasympathetic activity, relieving anxiety and depression. Stimulation of PC6 and ST36 alleviates visually induced nausea, increases gastric slow wave percentages, activates the vagus nerve to accelerate gastric emptying, and inhibits vasopressin-induced nausea and vomiting, as well as gastrointestinal symptoms like constipation [185, 186]. The gastrointestinal motility-promoting effect is believed to involve the 5-HT_4_ mechanism, while the antiemetic effect is thought to involve the 5-HT_3_ mechanism [187]. Acupoint stimulation regulates the DRG, SDH, and duodenal 5-HT system, decreasing 5-HT, serotonin, and dopamine levels, while also reducing circulating dopamine levels.

The HPA axis plays a significant role in the development of fatigue. The HPA axis includes CORT, ACTH, and CRH. Central inflammation can disrupt the function of the HPA axis, particularly affecting the synthesis and secretion of cortisol. Acupoint stimulation can increase serum levels of CORT and CRH, decrease the expression of ACTH, and alleviate fatigue symptoms [150, 188]. Depressive symptoms are not only highly related to the nervous system but are also associated with the immune and endocrine systems. Chronic neuroinflammation caused by the release of pro-inflammatory cytokines can lead to depression [189]. In patients with depression, serum levels of IgM and IgA are elevated, which may be indicative of systemic inflammatory responses of various natures. Endorphins can inhibit the functions of macrophages, natural killer cells, and T cells [190]. Acupoint stimulation induces the systemic release of corticosteroids while simultaneously upregulating biosynthetic pathways for endogenous opioid peptides including dynorphins, enkephalins, and endorphins in both the central and peripheral nervous systems. It also activates α7 nicotinic acetylcholine receptor, reducing the inflammatory cytokines produced via the vagus nerve, reversing IL-1β-related microglial activation induced by depression, and thereby inhibiting peripheral and central inflammatory responses to improve depressive symptoms [191–193].

Stimulation of ST36 can activate PROKR2^ADV^ neurons, thereby driving the vagal–adrenal anti-inflammatory axis. This leads to the release of norepinephrine, epinephrine, and dopamine from the adrenal chromaffin cells, which inhibits systemic inflammation induced by lipopolysaccharide, achieving a systemic anti-inflammatory effect [194]. The main underlying mechanisms are shown in Fig. 2.

As a comprehensive descriptive review, our primary objective was to establish a foundational framework for understanding the mechanisms of acupoint massage, rather than exhaustively cataloging the specific effects of individual acupoints, a task more appropriate for systematic reviews or dedicated databases. This review is based on the classification of nervous, endocrine and immune systems to synthesize generalizable mechanisms across various acupoint massage forms. We focused on commonly studied “hub” acupoints, as they are the most extensively researched and multifunctional, allowing us to highlight overarching principles that are applicable across different modalities. In summary, this part elucidates the multifaceted mechanisms underlying acupoint massage, highlighting its innovative potential in modulating neuro-endocrine-immune interactions. The findings underscore the importance of acupoint stimulation in promoting nerve regeneration, alleviating symptoms, and improving overall health, thereby offering significant academic value and practical implications for clinical practice.

## The future perspectives and conclusions

In exploring the potential of CAM, acupoint massage stands out as a promising therapy. With cancer’s global prevalence rising, the research value and prospects of acupoint massage in cancer treatment have gained prominence. To verify its efficacy and safety, there is an urgent need to combine rigorous and premium-quality basic mechanism research with large-scale, multi-disease, multi-age, disease stage and clinical evaluation of multi-centre RCT. These studies will elucidate underlying mechanisms, refine treatment protocols, and assess the therapy’s benefits in alleviating symptoms and enhancing quality of life. Through systematic evaluation, a robust theoretical framework can be established, facilitating methodical, superior-grade basic research advancements and achieving better cost-effectiveness in cancer treatment.

Gate control theory suggests pain can be offset by other sensations, such as pressure. Acupoint massage applies pressure to skin and soft tissues, activating pressure mechanoreceptors and allowing pleasurable signals to reach the brain faster than pain stimuli, inhibiting nociceptors [195, 196]. Precise application to acupoints or myofascial trigger points induces perceived comfort and symptomatic relief, mediated through pleasant tactile stimulation that engages somatosensory-affective integration pathways [197, 198]. However, Current evidence gaps persist regarding the specific neural circuitry underlying these therapeutic comfort responses, necessitating rigorous validation through further validation. Exploring these “pleasure neural circuits” will enhance our understanding and may advance acupoint massage, improving translational potential for enhancing treatment protocols and clinical outcomes. Additionally, recent studies findings have revealed that while the heat threshold increases in the elderly population, the pressure threshold remains relatively stable [199]. The discovery of threshold provides a theoretical basis and potential for the effective application of acupoint massage in older adults. Insomnia, anxiety, and depression are associated with specific pain predispositions, highlighting the importance of holistic pain management [200]. Acupoint massage alleviates various pains and improves mental disorder symptoms, enhancing holistic management by addressing both physiological and psychological factors.

Technological advancements are progressively transforming acupressure, shifting it from a therapist-dependent, hospital-based treatment to a precision-driven, accessible home healthcare model. AI-assisted acupoint localization now employs deep learning algorithms, which are trained on high-resolution anatomical datasets, historical clinical records, and advanced neural networks incorporating self-attention mechanisms to extract image features globally. These innovations, combined with human–computer interaction and novel positioning technologies, enable millimeter-level accuracy in acupoint recognition [201–205]. Additionally, the integration of particle swarm optimization-back propagation and backpropagation algorithms has significantly reduced force prediction errors in robotic systems, achieving single-digit force units and enhancing the consistency of acupoint application. Moreover, the integration of multimodal data, including individual pain thresholds, biomarker profiles, and genetic predispositions and further refines the learning models, enabling the adjustment of dynamic pressure parameters and treatment durations to optimize personalized therapeutic programs [206–208].

Wearable flexible massage devices, equipped with graphene-based skin sensors and shape-memory alloy actuators, facilitate real-time physiological monitoring while delivering adaptive mechanical stimulation. The convergence of 5G telemedicine platforms with IoT-connected massage systems enables remote supervision, allowing clinicians to modify treatment parameters via cloud-based AI analytics [204]. Theses technological synergy holds the potential for widespread adoption in low- and middle-income countries, offering cost-effective healthcare solutions as global populations age. These innovations are expected to position acupressure as a sustainable healthcare option, providing a feasible solution to address the challenges posed by aging populations and healthcare workforce shortages in the future [201, 209–211].

To fully harness the potential of acupoint massage therapy across various dimensions and types, future research must intensify its exploration of efficacy and mechanisms. This necessitates a detailed analysis of existing therapies and fosters interdisciplinary collaboration to re-evaluate traditional methods through innovative lenses. A rigorous examination of diverse acupoint massage forms and their clinical applications is vital for precise patient stratification, ensuring tailored treatment plans for individual conditions. Despite preliminary findings on clinical efficacy and potential mechanisms, this article focuses exclusively on mainstream applications and common mechanisms of action, aiming to provide a holistic view of acupoint massage’s overall development. Given the multifarious subfields and unique practices worldwide, defining and stratifying broader forms of acupoint massage remains essential, alongside exploring the neural circuits underlying pleasant touch transmission.

A thorough analysis of the diverse forms of acupoint massage and their applications in clinical practice is crucial for achieving precise stratification of patient groups, aiming to ensure that each patient receives the most appropriate treatment plan for their condition. Given the numerous subfields within acupoint massage and the unique practices of various countries, although preliminary results have been achieved in studying clinical efficacy and potential mechanisms, this article focuses solely on mainstream applications and common mechanisms of action. This choice aims to provide a general perspective on the overall development of acupoint massage.

But it is also necessary to pay attention to acupoint massage that, the heterogeneity of acupoint massage modalities, while reflecting rich traditional medical cultural and historical diversity, underscores the urgent need for international standardization to enhance reproducibility, comparability, and clinical applicability. Firstly, establish multidisciplinary frameworks. Collaborative efforts involving clinicians, researchers, and traditional medicine practitioners are essential to define core components of acupoint massage, such as, standardizing minimum and maximum pressure application times for specific conditions. These efforts should also include determining efficacy benchmarks, identifying validated outcome measures to assess therapeutic effects consistently across studies. At the same time, it should be noted that different acupoint forms are often related to traditional medical theories or cultures that have existed in different countries and regions for a long time, so it is necessary to harmonizing cultural and scientific perspectives, to create universally applicable protocols.

What’s more, leading by organizations such as the World Federation of Acupuncture-Moxibustion Societies and World Health Organization should prioritize to promote global collaboration, develop standardized nomenclature and classification systems of acupoint massage. Facilitating cross-cultural training programs to ensure consistent technique application. In addition, advancing research methodologies is crucial. Future studies should adopt more rigorous designs with standardized protocols, to validate the efficacy of specific and standardized modalities and assess their comparative effectiveness.

Future endeavours must develop robust methodologies to promote interdisciplinary integration, particularly with modern biological sciences. Studies on the neuro-endocrine-immune network and the identification of relevant biomarkers may provide valuable insights into the holistic nature of acupressure therapy. These findings could help elucidate the potential mechanisms underlying local, holistic, and point-specific treatments for a range of conditions. Additionally, they may shed light on factors such as age stratification and gender differences in the therapeutic response to the same disease. But a key point is the development of international standardization to enhance reproducibility, comparability and clinical applicability. At the same time, it will promote the rationalization and standardization of acupoint massage treatment and the patients’ belief and acceptance of acupoint massage therapy. The underlying mechanisms of these biological developments will facilitate the transition of acupoint massage from empirical to evidence-based practice. A comprehensive approach, considering both intrinsic and extrinsic factors of various techniques, may yield rational combinations, promising robust and enduring therapeutic outcomes. By addressing these challenges, acupoint massage can be advanced as a valuable complement to contemporary healthcare practices.

## Data Availability

All data used in this review are fully available in the public domain.

## Abbreviations

YLD: Years lived with disability
CAM: Complementary and alternative medicine
TCM: Traditional Chinese medicine
VAS: Visual analogue scale
CORT: Cortisol
RLS: Restless legs syndrome
PSQI: Pittsburgh sleep quality index
RCT: Randomized controlled trial
EMG: Electromyography
ATP: Adenosine triphosphate
ASIC3: Acid-sensing ion channel 3
TRP: Transient receptor potential
DRG: Dorsal root ganglia
SDH: Spinal dorsal horn
5-HT: 5-Hydroxytryptamine
nNOS: Nitric oxide synthase
HPA: Hypothalamic–pituitary–adrenal
CRH: Corticotropin-releasing hormone
ACTH: Adrenocorticotropic hormone
GABA: Gamma aminobutyric acid
HPO: Hypothalamic-pituitary-ovary
SIRT1: Skeletal muscle Sirtuin 1
IL-1β: Interleukin-1 beta
IL-2: Interleukin-2
IL-6: Interleukin-6
IL-4: Interleukin-4
ZO-1: Zonula occludens-1
TNF-α: Tumor necrosis factor-α
NF-κB: Nuclear factor kappa-light-chain-enhancer of activated B cells
ROS: Reactive oxygen species
BDNF: Brain-derived neurotrophic factor
Glu: Glutamate
RVM: Rostral ventral medulla
EAAT2: Excitatory amino acid transporter 2
